# Distinctions on the Importance of Age-Friendly Services by Old Age Groups: A Comparative Study Between the USA and the EU

**DOI:** 10.1093/geroni/igaa057.104

**Published:** 2020-12-16

**Authors:** David Bogataj, Marija Bogataj, Kathryn Hyer, Kathy Black

**Affiliations:** 1 University of Ljubljana, Ljubljana, Slovenia; 2 University of Ljubljana, Ljubljana, Ljubljana, Slovenia; 3 University of South Florida, Tampa, Florida, United States; 4 University of South Florida, Sarasota-Manatee, Sarasota, Florida, United States

## Abstract

Based on the initiative of the World Health Organization in 2007, when describing the global age-friendly cities by a guide to enhance active aging and the age-friendliness of communities, Black and Hyer (2019) presented generational distinctions on the importance of age-friendly community features by focusing efforts on the built, social, and service environment in USA. Their study aimed to examine the differential salience of community features by older generational age groups including Baby Boomers and younger persons 50 years and older, and older cohorts, born before and during WWII. They found that the Chi-square results indicate significant differences across the generational age groups in all domains. We wanted to compare the preference of the same age cohorts in Europe. The sizes of samples were half of the size of samples considered by Black and Hyer, therefore we used z+4 tests which also have shown the distinctions pertaining to preferences on housing and participation in social activities. The differences between ranking in importance of Age-Friendly Community Features by Older Age cohorts in USA and EU are presented and discussed.

